# Supplementary material for: Pathways to Increasing Adolescent Physical Activity and Wellbeing: A Mediation Analysis of Intervention Components Designed Using a Participatory Approach
Source: Int J Environ Res Public Health. 2020 Jan 7;17(2):390. doi: 10.3390/ijerph17020390 (PMC7014090; doi:10.3390/ijerph17020390)
Supplement: Supplementary file 1 [file ijerph-17-00390-s001.pdf]

ID

.

Thank you for agreeing to participate in the GoActive evaluation study.

In this booklet, we will be asking short questions about your experience with the GoActive programme.

Please complete all of the questions in this booklet.

- ✓ Please answer the questions as honestly and accurately as you can.
- ✓ Remember, this is not a test – there are no right or wrong answers!
- ✓ We will not tell anyone your answers unless we consider you or someone else to be at risk from harm, then we will tell one member of staff at your school.

1. Is the GoActive physical activity programme running at your school?

Tick the box to show whether you took part in the GoActive physical activity programme running at your school.

☐ No

☐ Yes

3. Please tell us whether you agree with the following statements

(Please tick one box for each item)

The **GoActive** study…	**Strongly agree**	**Slightly agree**	**Slightly disagree**	**Strongly disagree**	**Did not take part**
**a.** …is fun	☐	☐	☐	☐	☐
**b.** …encourages me to do more physical activity<br><br>*This statement refers to whether you felt <u>encouraged</u> to do more physical activity, not whether you actually did!*	☐	☐	☐	☐	☐
**c.** …allows me to try new activities	☐	☐	☐	☐	☐
**d.** …allows me to do physical activity with more people	☐	☐	☐	☐	☐
**e.** …takes/took up too much time	☐	☐	☐	☐	☐
**f.** …is boring	☐	☐	☐	☐	☐
**g.** …helps me make friends	☐	☐	☐	☐	☐
**h.** …gives me more confidence	☐	☐	☐	☐	☐
**i.** …introduces me to new activities	☐	☐	☐	☐	☐

2. How often do the following things happen since January?

(Please tick one box for each item)

	Never or hardly ever	Once or twice a week	Nearly every day	Every day
**a.** I tried new physical activities on my own	☐	☐	☐	☐
**b.** I asked friends at school to do physical activity with me	☐	☐	☐	☐
**c.** Friends at school asked me to do physical activity with them	☐	☐	☐	☐
**d.** I asked a parent/guardian to do physical activity with me	☐	☐	☐	☐
**e.** I asked a friend outside of school to do physical activity with me	☐	☐	☐	☐
**f.** I did the activity goals	☐	☐	☐	☐
**g.** I talked to friends about GoActive	☐	☐	☐	☐
**h.** I talked to my family about GoActive	☐	☐	☐	☐
**i.** I looked on the internet for activity ideas	☐	☐	☐	☐

4. Please let us know what you think of the different parts of the GoActive programme on a scale of 1 (did not like it) to 5 (liked it a lot).

Please circle the number that best describes what you think.

	Did not take part	Did not like it		Neither	Liked it a lot	
a. Working with mentors	1	2	3	4	5	6
b. Having year 9 leaders	1	2	3	4	5	6
c. Gaining individual points	1	2	3	4	5	6
d. The class competition	1	2	3	4	5	6
e. Suggesting new activities ourselves	1	2	3	4	5	6
f. Trying new activities	1	2	3	4	5	6
g. Individual prizes	1	2	3	4	5	6
h. T-shirts	1	2	3	4	5	6
i. Using tutor time	1	2	3	4	5	6
j. Choosing new activities	1	2	3	4	5	6
k. Working with form tutors	1	2	3	4	5	6
l. Trophies	1	2	3	4	5	6
m. Other (please state)________________________________	1	2	3	4	5	6

5. How likely are you to do the following on a scale of 1 (not likely) to 5 (very likely)?

Please circle the number that best describes what you think.

	Did not take part	Not likely		Fairly likely	Very likely	
a. Continue to do an activity I tried during GoActive	1	2	3	4	5	6
b. Join a new club or team relating to an activity I tried during GoActive	1	2	3	4	5	6
c. Encourage friends to do more physical activity	1	2	3	4	5	6
d. Encourage family members to do more physical activity	1	2	3	4	5	6

6. Would you be willing to be contacted about participating in a focus group (a group discussion) to discuss your GoActive experience?

These focus groups would be recorded and recordings would be made anonymous so that no participants can be identified from them. Unfortunately we will not be able to invite all students to a focus group.

☐ No

☐ Yes

7. Do you have any suggestions about how we could improve the GoActive programme?

____

____

____

8. Are/were you a Year 9 Leader during GoActive?

☐ Did not take part in GoActive

☐ No

☐ Yes

☐ *If yes, please write how many times you were a Year 9 leader during GoActive.*

9. If you are/were a Year 9 leader, please answer the following question.

Only answer this question if you ticked 'yes' to the question above.

**Being a Year 9 Leader for GoActive …**	**Strongly agree**	**Slightly agree**	**Slightly disagree**	**Strongly disagree**
**a.** …is fun	☐	☐	☐	☐
**b.** …improves my leadership skills	☐	☐	☐	☐
**c.** …takes up a lot of time	☐	☐	☐	☐
**d.** …is boring	☐	☐	☐	☐
**e.** …gives me more confidence	☐	☐	☐	☐
**f.** …is a lot of work	☐	☐	☐	☐
**g.** …is easy	☐	☐	☐	☐

10. Do you have any comments about being a leader?

____
____
____

Thank you for completing the questionnaire – we really appreciate your time!
